# MIF Inhibition by ISO-1 Decreased Autophagic Activity in Primary Astrocytes During Cobalt Chloride-Induced Hypoxia

**DOI:** 10.3390/cimb46120813

**Published:** 2024-11-29

**Authors:** Ji-Hyun Park, Hye-Ji Cho, Dae-Yong Song, Jung-Ho Lee, Hong-Il Yoo

**Affiliations:** 1 Department of Anatomy and Neurosciences, School of Medicine, Eulji University, Daejeon 34824, Republic of Korea; qkrwlgus929@naver.com (J.-H.P.); dysong@eulji.ac.kr (D.-Y.S.); 2 Department of Pharmacology, School of Medicine, Eulji University, Daejeon 34824, Republic of Korea; 20221685@eulji.ac.kr

**Keywords:** primary astrocytes, hypoxia, MIF, autophagy

## Abstract

Ischemic stroke is a leading contributor to death and disability worldwide, driving extensive research into pharmacological treatments beyond thrombolysis. Macrophage migration inhibitory factor (MIF), a cytokine, is implicated in several pathological conditions. In this study, we examined the effects of MIF on autophagy in astrocytes under the condition of chemical hypoxia. Primary astrocytes were treated with cobalt chloride, a well-known drug for inducing chemical hypoxia, followed by Western blot analyses to assess the HIF-1α, MIF, and LC3 protein levels along with a CCK assay. Additionally, cobalt chloride-treated astrocytes were co-treated with the MIF inhibitor ISO-1, and Western blot analyses were performed for MIF and LC3. Cell viability was evaluated using the CCK assay in astrocytes treated with cobalt chloride and ISO-1, with additional rapamycin treatment. Our results show that ISO-1 reduced LC3-II levels in astrocytes exposed to high concentrations of cobalt chloride (1000 μM) for 6 h. Moreover, rapamycin decreased cell viability in astrocytes treated with both 1000 μM cobalt chloride and ISO-1. Our data suggest that MIF plays a role in inducing autophagy in astrocytes under hypoxic conditions and is involved in the regulation of autophagic activity.

## 1. Introduction

Ischemic stroke is a leading contributor to death and disability worldwide [1]. Despite extensive research on therapeutic approaches, thrombolysis remains the only pharmacologic treatment with proven efficacy. However, thrombolytic therapy is constrained by a restricted time frame for administration following symptom onset and contraindications, rendering many ischemic stroke patients ineligible [1]. As a result, vigorous ongoing research is being conducted with the aim of identifying alternative treatments with neuroprotective effects, either independently or in combination with thrombolysis.

Astrocytes—the most abundant glial cells—have traditionally received less attention in brain disease research than neurons, but recent research indicates that they are critically involved in disorders including Alzheimer’s disease (AD) and Parkinson’s disease (PD) [2]. Normally, astrocytes maintain neuronal homeostasis by recycling neurotransmitters [3], regulating synapses [4], and supporting the blood–brain barrier [5]. However, under pathological conditions such as inflammation or trauma, astrocytes become reactive and contribute to central nervous system inflammation through interactions with microglia [2]. Reactive astrocytes also play a dual role in brain ischemia: they can protect neurons from oxidative stress and reduce cerebral edema, but in severe cases, their impaired glutamate uptake may lead to neuronal death, making them a “double-edged sword” [6].

One of the most widely used in vitro models for studying the pathology of brain ischemia involves treating cells with cobalt chloride, a hypoxia mimetic agent [7]. Hypoxia-inducible factor (HIF)-1α acts as a master regulator that maintains cellular homeostasis under hypoxic conditions by reprogramming glycolytic metabolism and by reducing the production of mitochondrial reactive oxygen species [8]. Cobalt chloride inhibits the activity of prolyl hydroxylase, which, in response to oxygen levels, mediates the proteasomal degradation of HIF-1α [9]. Through this mechanism, cobalt chloride stabilizes HIF-1α, thereby mimicking hypoxic conditions. In this study, we utilized an astrocyte model treated with cobalt chloride.

Ischemic injury has been found to trigger autophagy in astrocytes both in vitro and in vivo [10,11]. However, the exact signaling pathways that control the balance between autophagy and apoptosis in astrocytes as ischemic damage intensifies remain debated. Some research has suggested that blocking autophagy in astrocytes reduces their reactive gliosis and reduces apoptosis [10,12], while other studies have indicated that inhibiting autophagy decreases astrocyte survival and raises the number of apoptotic cells [13,14]. Autophagy is a critical cellular mechanism that maintains homeostasis and supports cell viability by facilitating the bulk degradation of intracellular proteins and organelles. A key step in the formation of the autophagosome—the structure that engulfs intracellular components for degradation—is the modification of light chain 3 (LC3), converting LC3-I into its lipidated form, LC3-II [15].

Macrophage migration inhibitory factor (MIF) is a cytokine produced by various cell types, including hepatocytes, endothelial cells, and neurons [16,17,18]. MIF has been characterized as an immunoregulatory molecule and molecular chaperone [19,20], as well as a tumorigenic factor [21]. From the perspective of autophagy, specifically mitophagy, the secretion of MIF can be regulated in macrophages in response to mitochondrial reactive oxygen species (ROS) release [22]. Furthermore, MIF is known to function as an autophagic regulator [23]. Given that autophagy can either promote or inhibit cell death depending on the cellular environment and stimuli, there is limited research on the role of MIF in autophagy under hypoxic conditions in the central nervous system, particularly in astrocytes.

We sought to explore how MIF regulates astrocyte autophagy in relation to the intensity and duration of hypoxia, and how these changes influence the response of primary astrocytes to hypoxic stress.

## 2. Materials and Methods

### 2.1. Preparation of Mixed Cortical Cell Isolation

We performed mixed cortical cell isolation using brains from newborn rats. Timed pregnant (TP) female SD rats were purchased from Samtako BioKorea (Osan, Republic of Korea). We conducted all animal experiments in accordance with approved protocols and guidelines established by the Eulji University Institutional Animal Care and Use Committee (Approval number: EUIACUC22-05). Rat pups aged between P3 and P4 were disinfected with 70% ethanol spray and then sacrificed by decapitation using fine scissors. The scalp and cranium were cut to expose the entire brain. The cerebrum, following the removal of the olfactory bulb, cerebellum, and brainstem, was transferred to a dish containing ice-cold Hank’s Balanced Salt Solution (HBSS; Gibco, Waltham, MA, USA). The cerebral cortices were isolated from the cerebrum, and the meninges were carefully removed under a microscope to prevent contamination by meningeal fibroblasts. The cortices were subsequently placed into a separate dish containing chilled HBSS, with three rat pups’ brains (six cortical pieces) pooled per dish. The cortices were chopped into fine pieces using a sharp blade.

Under sterile conditions, we transferred the cortical pieces into 50 mL Falcon tubes, and HBSS and 2.5% trypsin (10×) (Thermo Fisher Scientific, Waltham, MA, USA) were added to make a total volume of 25 mL. The tubes were placed in a water bath at 37 °C for 30 min of incubation, after which the mixed cortical cells were mechanically dissociated via vigorous pipetting in 10 mL of astrocyte culture medium (Dulbecco’s Modified Eagle Medium; WELGENE, Gyeongsan, Republic of Korea) containing 10% fetal bovine serum (FBS001-HI, Neuromics, Edina, MN, USA) and 1% Penicillin/Streptomycin (CA005-010, GenDEPOT, Baker, TX, USA). After centrifugation, we seeded the mixed cortical cells into poly-L-lysine-coated T75 flasks and cultured them in media, which was changed every two days. The morphology of the cells and confluence were checked daily under a microscope.

### 2.2. Purification of Primary Astrocyte Cultures

After 7 to 8 days, once the mixed cortical cells reached confluence, the T75 flasks were shaken at 180 rpm for 30 min using an orbital shaker (JSSI-350T, JSR, Gongju, Republic of Korea) to eliminate microglia. Following a change of media, oligodendrocytes were detached by agitating the flasks at 240 rpm for 6 h. The remaining astrocytes were detached using trypsin and expanded in astrocyte culture media until they reached 70–80% confluence in a 90 mm dish. An immunofluorescence analysis revealed that the astrocyte population had a purity of approximately 95% under these conditions, based on staining with anti-Iba1 for microglia, anti-OLIG2 for oligodendrocytes, and anti-GFAP for astrocytes.

### 2.3. Chemical-Induced Hypoxia

For chemical-induced hypoxia, cobalt chloride (CoCl_2_; C8661, Sigma-Aldrich, St. Louis, MO, USA) was diluted in the experimental medium at working concentrations of 100 to 1000 μM [24,25,26]. ISO-1 (#475837, EMD Millipore, Burlington, VT, USA), the MIF antagonist, was also used to investigate the role of MIF under hypoxic conditions.

### 2.4. CCK Assay

Primary astrocytes were plated at 1 × 10^5^ cells/plate in a 96-well cell culture plate and incubated in a 5% CO_2_ incubator at 37 °C for 24 h. Subsequently, the cells were treated with various combinations of cobalt chloride, ISO-1, and rapamycin (553210, Sigma-Aldrich, USA) for either 24 or 48 h. After treatment, 10 μL of CCK-8 reagent (Dojindo, Kumamoto, Japan) was added to each well, followed by incubation in the dark for 2 h. Absorbance at 450 nm was then measured using a microplate photometer (Thermo Scientific, Waltham, MA, USA).

### 2.5. Western Blot

Proteins were extracted from astrocyte cultures using RIPA lysis buffer (Thermo Scientific) supplemented with protease and phosphatase inhibitors. Total protein concentrations were determined with a BCA protein assay kit (Thermo Scientific). Protein samples (5 μg each) were separated via SDS-PAGE and transferred onto nitrocellulose (NC) membranes. The membranes were blocked for 1 h in TBS containing 4% BSA and 0.1% Tween-20 (TBST), followed by overnight incubation at 4 °C with primary antibodies, including anti-β-actin (1:2000, sc47778, SCBT, Dallas, TX, USA), anti-HIF-1α (1:300, ab179483, Abcam, Cambridge, UK), and anti-LC3 (1:1000, #4108, CST, Danvers, MA, USA). After three 10 min washes in TBST, membranes were treated with appropriate secondary antibodies (1:2000, CST, USA) such as goat anti-mouse or anti-rabbit antibodies. The blots were visualized using an enhanced chemiluminescence (ECL) system (Amersham Pharmacia Biotech, Chicago, IL, USA) and captured with the Chemi-Doc™ Touch Imaging System (Bio-Rad, Richmond, CA, USA). In the LC3 Western blot, LC3-I (upper band) and LC3-II (lower band) were detected, and we measured the band intensity of LC3-II, which is known as an autophagosomal marker [15].

### 2.6. Statistical Analysis

The experiments were conducted a minimum of three times. The results are presented as mean values ± standard deviation (SD) and were analyzed using the GraphPad Prism 8 software package (GraphPad Software, Boston, MA, USA). Differences among multiple groups were assessed using one-way ANOVA followed by Tukey’s post hoc test. A *p*-value below 0.05 was considered statistically significant in all evaluations.

## 3. Results

### 3.1. Cobalt Chloride Treatment Induced HIF-1α Expression and Cytotoxicity in Primary Astrocyte Culture

**Figure 1:**
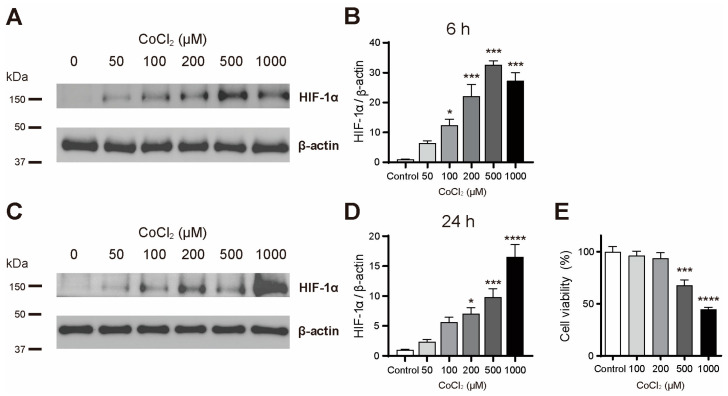
Cobalt chloride treatment induced increases in HIF-1α levels and cytotoxicity in primary astrocytes. (**A**) Representative of the HIF-1α Western blot after 6 h of the cobalt chloride treatment. (**B**) Quantification of HIF-1α protein levels in astrocytes after 6 h of the cobalt chloride treatment (n = 5). (**C**) Representative of the HIF-1α Western blot after 24 h of the treatment. (**D**) Quantification of HIF-1α protein levels in astrocytes after 24 h of the cobalt chloride treatment (*n* = 5). (**E**) Quantification of CCK-8 assay results after 48 h treatment (*n* = 8). Data are presented as the mean ± SD. * *p* < 0.05, *** *p* < 0.001, and **** *p* < 0.0001 compared to the control (vehicle group).

First, primary astrocytes were exposed to different concentrations of cobalt chloride (50, 100, 200, 500, and 1000 μM)—a hypoxia-mimicking agent—for 6 and 24 h to examine the changes in HIF-1α levels using Western blot analysis. HIF-1α protein levels increased in a dose-dependent manner across most concentrations after cobalt chloride treatment for both 6 and 24 h. After 6 h, concentrations above 100 μM resulted in a statistically significant increase in HIF-1α protein levels (Figure 1A,B). After 24 h, a significant increase in HIF-1α protein levels was observed at concentrations above 200 μM (Figure 1C,D). These findings indicate that cobalt chloride effectively triggers a hypoxic response in primary astrocytes. Following this, astrocytes were treated with 0, 100, 200, 500, and 1000 μM of cobalt chloride for 48 h, and cell viability was assessed using a CCK assay. The results demonstrated that concentrations of 500 and 1000 μM significantly reduced astrocyte viability (Figure 1E), confirming the cytotoxic effect of the cobalt chloride treatment.

### 3.2. Cobalt Chloride Treatment Altered Autophagic Activity in Primary Astrocyte Culture

**Figure 2:**
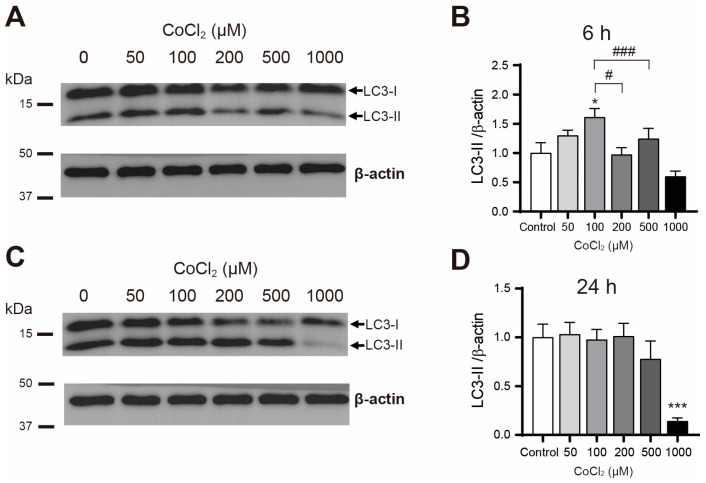
Cobalt chloride treatment changed the LC3-II levels in primary astrocytes. (**A**) Representative of the LC3-II Western blot after 6 h of the cobalt chloride treatment. The positions of the LC3-I and LC3-II bands are indicated with arrows. (**B**) Quantification of LC3-II protein levels in astrocytes after 6 h of the cobalt chloride treatment (*n* = 5). (**C**) Representative of the LC3-II Western blot after 24 h of treatment. The positions of the LC3-I and LC3-II bands are indicated with arrows. (**D**) Quantification of LC3-II protein levels in astrocytes after 24 h of the cobalt chloride treatment (*n* = 5). Data are presented as the mean ± SD. * *p* < 0.05, *** *p* < 0.001 compared to the control (vehicle group). # *p* < 0.05, ### *p* < 0.001 compared to the 100 μM treatment group.

To investigate the effect of cobalt chloride on autophagic activity, primary astrocytes were treated with 0, 50, 100, 200, 500, and 1000 μM of cobalt chloride for 6 and 24 h, followed by a Western blot analysis of LC3 protein levels. After 6 h, cobalt chloride significantly increased LC3-II levels at 100 μM (Figure 2A,B). However, at concentrations of 200 μM and above, LC3-II levels did not increase and were significantly lower at 200 and 500 μM compared to the 100 μM treatment (Figure 2A,B). After 24 h, LC3-II levels were significantly reduced at 1000 μM (Figure 2C,D), suggesting that while cobalt chloride enhances autophagic activity at lower concentrations, this effect diminishes at higher concentrations.

### 3.3. Cobalt Chloride Treatment Increased MIF Levels in Primary Astrocyte Culture

**Figure 3:**
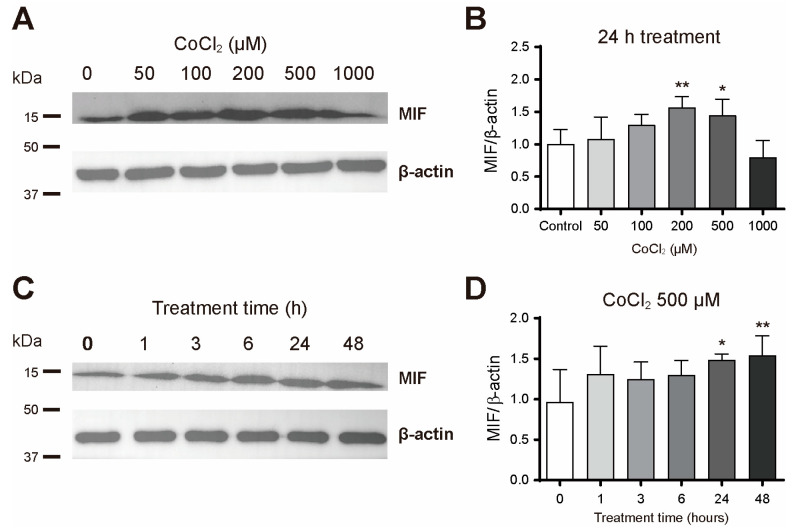
Cobalt chloride treatment increased the MIF levels in primary astrocytes. (**A**) Representative of the MIF Western blot after 24 h of the cobalt chloride treatment. (**B**) Quantification of MIF protein levels in astrocytes after 24 h of the cobalt chloride treatment (*n* = 5). (**C**) Representative of the MIF Western blot after 0, 1, 3, 6, 24, and 48 h of the 500 μM cobalt chloride treatment. (**D**) Quantification of MIF protein levels in astrocytes after various durations of the cobalt chloride treatment (*n* = 5). Data are presented as the mean ± SD. * *p* < 0.05, ** *p* < 0.01, compared to the control (vehicle group).

To assess the effects of cobalt chloride on MIF protein levels, we performed a Western blot analysis after treatment with varying concentrations (50, 100, 200, 500, and 1000 μM) and durations (1, 3, 6, 24, and 48 h). When primary astrocytes were treated with different concentrations of cobalt chloride for 24 h, statistically significant increases in MIF levels were observed at 200 and 500 μM (Figure 3A,B). Treatment with 500 μM cobalt chloride led to a statistically significant increase in MIF levels in both the 24 h and 48 h treatment groups (Figure 3C,D). These findings demonstrate that, under relatively high concentrations (200 and 500 µM) and extended exposure times (24 and 48 h), cobalt chloride-induced chemical hypoxic conditions increase MIF protein levels.

### 3.4. MIF Inhibition by ISO-1 Reduced Autophagic Activity in Cobalt Chloride-Treated Primary Astrocytes

**Figure 4:**
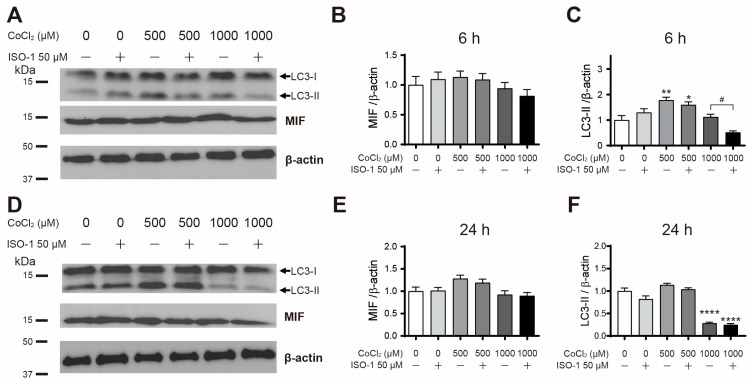
MIF inhibitor ISO-1 cotreatment altered LC3-II levels of cobalt chloride-treated primary astrocytes. (**A**) Representative of the MIF and LC3-II Western blot after 6 h of the ISO-1 and cobalt chloride co-treatment. The positions of the LC3-I and LC3-II bands are indicated with arrows. (**B**) Quantification of MIF protein levels in astrocytes after 6 h of the treatment (*n* = 5). (**C**) Quantification of LC3-II protein levels in primary astrocytes after 6 h of the treatment (*n* = 5). (**D**) MIF and LC3-II Western blot after 24 h of the ISO-1 and cobalt chloride co-treatment. The positions of the LC3-I and LC3-II bands are indicated with arrows. (**E**) Quantification of MIF protein levels in astrocytes after 24 h of the treatment (*n* = 5). (**F**) Quantification of LC3-II protein levels in primary astrocytes after 24 h of the treatment (*n* = 5). Data are presented as the mean ± SD. * *p* < 0.05, ** *p* < 0.01, **** *p* < 0.0001 compared to the control (vehicle group). # *p* < 0.05 between 1000 μM cobalt chloride only treatment group and 1000 μM cobalt chloride and ISO-1 50 μM co-treatment group.

To evaluate the effect of MIF inhibition on cobalt chloride-treated astrocytes, cells were co-treated with the MIF inhibitor ISO-1 and cobalt chloride (500, 1000 μM) for 6 and 24 h. Although MIF protein levels did not show statistically significant differences among all groups at 6 h of treatment, at 24 h, there was a slight increase in MIF protein levels in the group treated with 500 μM cobalt chloride alone and in the group co-treated with 500 μM cobalt chloride and ISO-1; however, this increase did not reach statistical significance (Figure 4A,B,D,E). These results suggest that MIF protein levels are not significantly affected by ISO-1 treatment under hypoxic conditions. Next, to investigate the effect of MIF inhibition on autophagy activity, LC3-II protein levels were determined by Western blotting. MIF inhibition with ISO-1 tended to decrease LC3-II levels in cobalt chloride-treated astrocytes. Specifically, LC3-II levels were significantly lower in the group co-treated with ISO-1 and high-concentration cobalt chloride (1000 μM) for 6 h, when compared to the group treated with cobalt chloride alone (Figure 4A,C). On the other hand, in the 24 h treatment group, LC3-II expression decreased compared to the control when treated with 1000 μM cobalt chloride, regardless of the presence of ISO-1 (Figure 4D,F). These results indicate that MIF inhibition reduces autophagy activity under relatively short-term hypoxic conditions but does not significantly affect autophagy under severe hypoxic conditions or prolonged hypoxia.

### 3.5. Rapamycin Treatment Reduced Cell Viability in ISO-1 and Cobalt Chloride Co-Treated Primary Astrocytes

**Figure 5:**
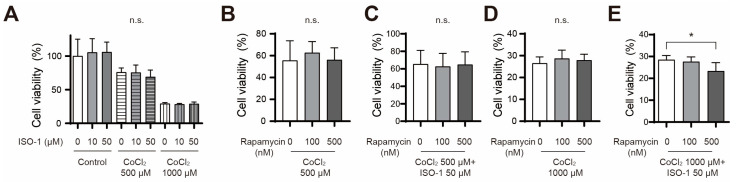
The addition of rapamycin to ISO-1 and cobalt chloride co-treated primary astrocytes reduced cell viability. (**A**) Quantification of CCK-8 assay results after 48 h ISO-1 and cobalt chloride co-treatment (*n* = 8). (**B**) CCK-8 assay results after 48 h rapamycin and 500 μM cobalt chloride co-treatment (*n* = 8). (**C**) CCK-8 assay results after 48 h rapamycin, 500 μM cobalt chloride, and 50 μM ISO-1 co-treatment (*n* = 8). (**D**) CCK-8 assay results after 48 h rapamycin and 1000 μM cobalt chloride co-treatment (*n* = 8). (**E**) CCK-8 assay results after 48 h rapamycin, 1000 μM cobalt chloride, and 50 μM ISO-1 co-treatment (*n* = 8). Data are presented as the mean ± SD. * *p* < 0.05 between 1000 μM cobalt chloride and 50 μM ISO-1 co-treatment group and rapamycin 500 nM, 1000 μM cobalt chloride, and 50 μM ISO-1 co-treatment group.

To assess the cell viability, a CCK assay was conducted in primary astrocytes co-treated with ISO-1 (10 and 50 μM) and cobalt chloride (500 and 1000 μM). No statistically significant difference in cell viability was observed between the group co-treated with ISO-1 and cobalt chloride and the group treated with cobalt chloride alone (Figure 5A). To further explore the role of ISO-1 in autophagy regulation, astrocytes co-treated with cobalt chloride and ISO-1 were also exposed to rapamycin, an mTOR inhibitor known to promote autophagy. Co-treatment with rapamycin, along with 500 μM cobalt chloride, 500 μM cobalt chloride and ISO-1, or 1000 μM cobalt chloride, did not result in any significant change in cell viability (Figure 5B–D). Interestingly, the assay results showed a decrease in cell viability in the group that received additional treatment with rapamycin (500 nM) compared to the group treated with only cobalt chloride (1000 μM) and ISO-1 (Figure 5E).

## 4. Discussion

We demonstrated for the first time that ISO-1 treatment reduces autophagic activity in cobalt chloride-treated astrocytes. Furthermore, we found that rapamycin treatment decreases cell viability in astrocytes co-treated with ISO-1 and cobalt chloride.

In this study, we used cobalt chloride to induce chemical hypoxia in primary astrocytes, as it is widely employed to create chemical hypoxia models in settings where actual hypoxic conditions are difficult to replicate. Our goal was to investigate the effects of MIF inhibition via ISO-1 under both mild and severe hypoxic stress conditions induced using cobalt chloride. The precise role of autophagy in ischemic stroke remains uncertain. Autophagy, which can be triggered by exogenous or endogenous stress, acts as a double-edged sword: while it may support cell survival under mild stress, excessive autophagy under prolonged or severe stress can result in cell death through apoptosis or necrosis [27]. Thus, modulating the degree of chemical hypoxia to observe changes in the role of autophagy in response to hypoxia could offer a more comprehensive understanding of its underlying mechanisms. Due to the advantage of cobalt chloride regarding its ability to impose various levels of stress in cells, we utilized cobalt chloride-treated astrocytes in our study.

Several previous studies have reported that the cobalt chloride-induced stabilization of HIF-1α is associated with increased transcriptional activity of HIF-1α in astrocytes. Karovic et al. demonstrated that treatment with cobalt chloride in primary mouse astrocytes led to an increase in HIF-1α protein levels, which was accompanied by the upregulation of the mRNA expression of Nip3—a well-characterized HIF-1α target [24]. Similarly, Rempe et al. found that the genetic deletion of HIF-1α in primary mouse astrocytes significantly reduced the expression of HIF-1α targets (Glut-1, HKII, Nip3, and Nix) under hypoxia—a condition known to induce HIF-1α stabilization [28]. Zheng et al. showed that beta-amyloid treatment in primary mouse astrocytes decreased HIF-1α protein levels, while co-treatment with beta-amyloid and GLP-1 restored HIF-1α protein levels. Notably, the mRNA expression of several glycolytic enzymes mirrored the changes in HIF-1α protein levels [29]. Furthermore, Allen et al. reported that primary human astrocytes cultured under hypoxic conditions exhibited the upregulation of genes associated with glycolytic functions, including phosphoglycerate kinase 1 (PGK1), pyruvate kinase (PKM), and pyruvate dehydrogenase kinase isozyme 3 (PDK3), when compared to cells cultured under normoxic conditions [30].

Previous studies have demonstrated that hypoxia increases MIF protein levels across various cell types, including astrocytes [31,32,33]. A recent study reported that HIF-1α promoted MIF production in astrocytes in an animal model of spinal cord injury [33]. The increase in MIF levels observed in our experiments is likely a response to the elevated HIF-1α levels induced by cobalt chloride. MIF is known to induce autophagy in various cell types [34,35,36,37]. The role of MIF in autophagy can be context-dependent, offering benefits under certain pathological conditions while exacerbating others; for instance, MIF-induced autophagy in endothelial cells is implicated in mediating vascular leakage during inflammatory shock [36]. Additionally, the genetic deletion of MIF has been shown to aggravate cardiac hypertrophy in a moderate pressure overload animal model [37]. One study showed that MIF overexpression elevated LC3-II levels in a Parkinson’s disease animal model, suggesting a neuroprotective effect [34]. Our data demonstrated that the inhibition of MIF using ISO-1 reduced autophagic activity.

In this study, we found that inducing autophagy with rapamycin in primary astrocytes exposed to both severe hypoxia caused by a high concentration (1000 μM) of cobalt chloride and MIF inhibition via ISO-1 treatment resulted in decreased cell viability. Both severe ischemia and ISO-1 treatments reduced autophagy in primary astrocytes; however, this combined reduction in autophagy did not impact cell viability. Under these conditions, rapamycin-induced autophagy led to a decline in cell viability. As previously noted, excessive autophagy under severe ischemic conditions can promote cell death. A previous study demonstrated that the genetic inhibition of MIF did not directly influence AMPK or mTOR activation but did enhance changes in their activation by other factors [38]. This suggests that following MIF inhibition, rapamycin-induced mTOR inhibition may have triggered excessive autophagy, leading to cytotoxicity. Nonetheless, further studies are needed to elucidate the specific mechanisms by which hypoxia-induced astrocytic autophagy influences the pathology of cerebral ischemia; additionally, future research should focus on how MIF overexpression affects the balance between the autophagy and apoptosis of astrocytes under hypoxic conditions in order to elucidate the signaling pathway of MIF, a multi-faceted cytokine considered one of the potential therapeutic targets for ischemia. In actual stroke pathology, ischemic stress exhibits varying intensities depending on the anatomical relationship to the occluded vascular region, and the cellular composition of the brain is highly heterogeneous. Our in vitro experimental conditions did not fully replicate the complex cellular environment of the brain, which comprises diverse cell types, such as neurons, various subtypes of reactive astrocytes, and microglia. Moreover, complications associated with thrombolytic therapy and reperfusion injury further contribute to the complexity of stroke-related mechanisms; however, these aspects were also beyond the scope of this study. Future studies using animal models of stroke are needed to elucidate the roles of MIF in stroke pathology and to explore its potential therapeutic implications.

## Data Availability

Data are contained within the article.
